# “The uncharted waters of completely not knowing what’s next”: rare cancer patients’ experiences regarding navigating uncertainty

**DOI:** 10.1007/s00520-026-11331-4

**Published:** 2026-10-15

**Authors:** Tamsin Farrugia, Saskia F. A. Duijts, Carlene Wilson, Christine Cockburn, Laura Hemming, Evelien Spelten

**Affiliations:** 1 Violet Vines Marshman Centre for Rural Health Research, Rural Health School, La Trobe University, Bendigo, VIC 3552 Australia; 2 Department of Research and Development, Netherlands Comprehensive Cancer Organisation (Integraal Kankercentrum Nederland, IKNL), Utrecht, The Netherlands; 3 Department of Medical Psychology, Amsterdam University Medical Center, Location Vrije Universiteit, Amsterdam, The Netherlands; 4 Cancer Center Amsterdam, Cancer Treatment and Quality of Life, Amsterdam, The Netherlands; 5 Rare Cancers Australia, Bowral, NSW 2576 Australia; 6 Melbourne School of Population and Global Health, The University of Melbourne, Melbourne, VIC Australia

**Keywords:** Rare cancer, Illness uncertainty, Mishel’s uncertainty in illness theory, Supportive care, Oncology

## Abstract

**Purpose:**

A rare cancer diagnosis can cause a great deal of uncertainty. However, rare cancer survivors’ experience with illness uncertainty is underreported in the literature. This study aimed to explore how people diagnosed with a rare cancer experience and manage uncertainty throughout their cancer experience.

**Methods:**

A secondary analysis of interview data was undertaken. Data were obtained from semi-structured interviews conducted with 20 rare cancer survivors between November 2023 and February 2024. Interviews were recorded and transcribed verbatim. Data were analysed in terms of how participants experienced uncertainty, as described in Mishel’s reconceptualised uncertainty in illness theory (e.g., forms, sources and influences on illness uncertainty). Deductive thematic analysis was applied, using NVivo 15 software.

**Results:**

Illness uncertainty was most likely experienced in the form of ambiguity or the receipt of inadequate information. The primary source of illness uncertainty arose from event unfamiliarity. The quality and extent of information provision from healthcare providers had the greatest influence over reported illness uncertainty. Nonetheless, social support could also reduce perceptions of uncertainty, particularly amongst those who had completed treatment. Participants adopted various coping strategies in response to uncertainty, such as information seeking, with adaptation occurring over time.

**Conclusion:**

Rare cancer survivors experience illness uncertainty throughout their cancer trajectory. In line with Mishel’s theory, future research should develop interventions that reduce ambiguity, enhance healthcare provider awareness of the importance of information and its impact on patient wellbeing, and support adaptation. This will improve informational and psychosocial outcomes, and reduce uncertainty, for rare cancer patients.

## Background

Illness uncertainty refers to the inability to make sense of an illness-related event [1]. Coping with uncertainty about the future is one of the greatest unmet needs for cancer survivors [2] and is widely acknowledged as a significant source of psychological distress, depression and anxiety, and compromised quality of life [3–6]. When diagnosed with a rare cancer, rarity-related challenges can magnify uncertainty and add to the already heavy burden faced by anyone confronting a cancer diagnosis.

Rare cancers are defined as those cancer types with an incidence of less than 6 cases per 100,000 people per year, accounting for 12% of all cancers diagnosed in Australia in 2025 [7]. Rare cancer patients are likely to experience a lack of information about their specific cancer type, reduced access to clinical expertise and adequate treatment options [8, 9] and a complex and less predictable disease progression compared to common cancer patients [10, 11]. Uncertainty increases when there is ambiguity about the state of the illness, inconsistent or inadequate information, complexity in treatment and/or when the disease has an unpredictable nature [1, 12]. Therefore, rare cancer patients experience conditions that, theoretically, increase uncertainty. The literature in this space, however, is limited.

**Fig. 1:**
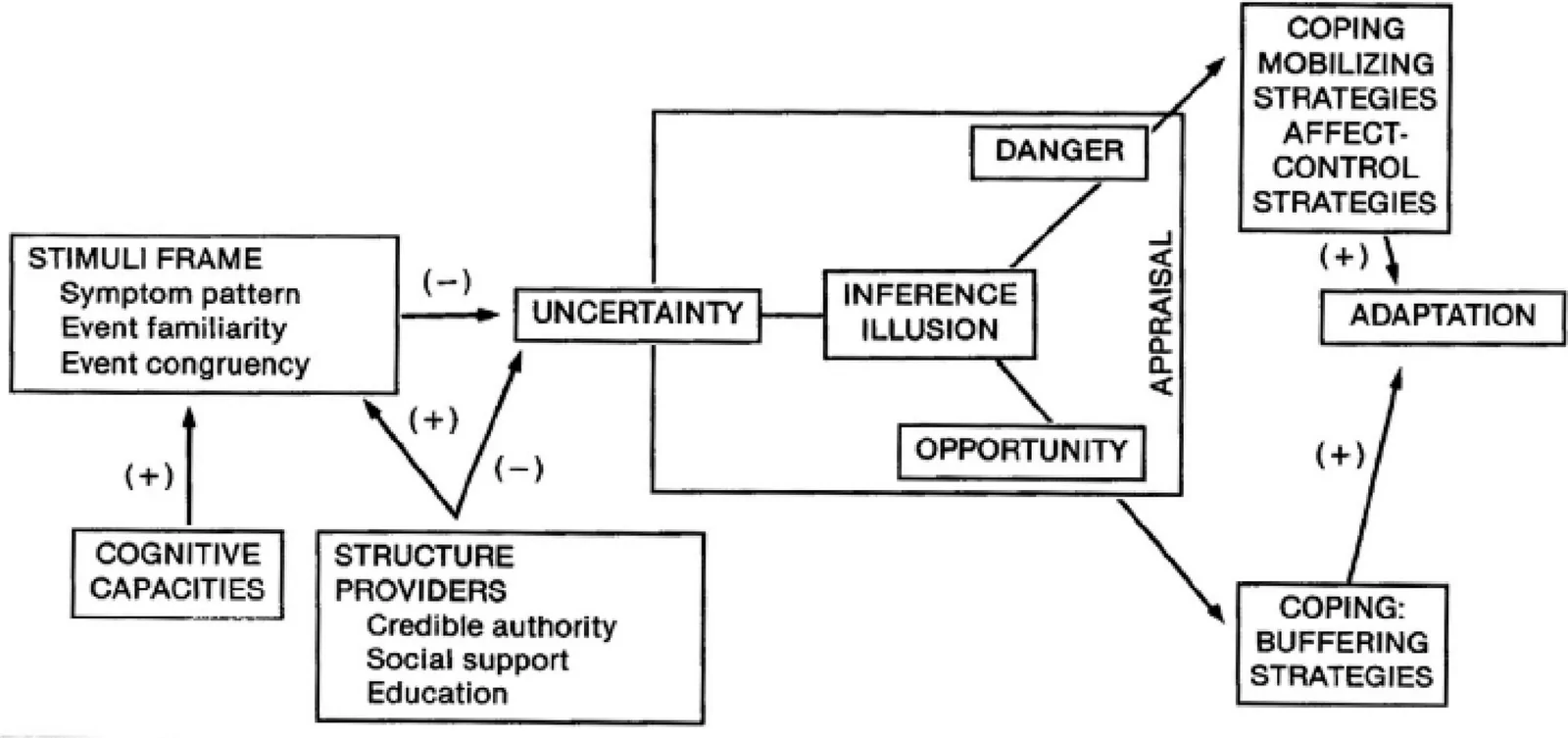
Mishel’s reconceptualised uncertainty in illness theory

Mishel’s reconceptualised uncertainty in illness theory (RUIT) [12] (Fig. 1) is one of several theories [13, 14] developed to explore chronic disease patients’ experiences with illness uncertainty, outlining the antecedents, influencers, appraisal and coping mechanisms, including effective adaptation, for uncertainty [12]. According to the RUIT, illness uncertainty is preceded by the ‘stimuli frame’, otherwise known as the sources of uncertainty that include the patterns of symptoms, event (un)familiarity and event congruence (i.e. the consistency between a person’s expectations and their actual experience). The person’s cognitive capacity to process information and ‘structure providers’ (i.e. credible authority, social support and education) influence whether the uncertainty is appraised as either negative or positive (i.e. as perceptions of danger or opportunity). Depending on how the uncertainty is appraised, coping strategies are adopted. Over time, patients learn to adapt to their illness uncertainty through acceptance and probabilistic thinking, embracing uncertainty as inherent to chronic illness [12].

In the context of rare cancer, vague and persistent symptoms, multiple hospital visits, receiving an incorrect diagnosis and experiencing different treatment experiences to what was expected [15] are examples of the ‘stimuli frame’, explaining the symptom pattern, event familiarity and event congruence, respectively.

Previous studies have demonstrated the application of Mishel’s RUIT to people with a chronic disease [16]; however, exploration of its applicability to people living with a rare cancer has not been conducted. Of the research published on rare cancer uncertainty, this has focused on quantitative studies with people diagnosed with head and neck cancers (HNCs) and their ‘uncertainty about the future’ [8]. The extent to which rare cancer patients experience and manage illness uncertainty, and how uncertainty manifests at different stages of the cancer path, remains underexplored. In our previous qualitative study, we explored the perspectives of rare cancer survivors and healthcare professionals on the challenges of information access and provision [17]. In this context, all survivors highlighted their experience of illness uncertainty. Therefore, the aim of the current paper was to explore how people diagnosed with a rare cancer experience and manage uncertainty throughout their cancer experience, applying Mishel’s RUIT as a framework for analysis.

## Methods

### Design

We conducted a secondary analysis of data from our previous qualitative study, which explored rare cancer survivors’ and healthcare professionals’ preferences for information provision. In this current analysis, we focus on the survivor interview data only. The original study design, participant recruitment procedure and data collection procedure have been described in detail previously [17]. The study was approved by La Trobe University’s Human Ethics Committee (HEC 23234), which permitted the publication of multiple research articles reporting the study findings. All researchers involved in the analysis and reporting of this secondary analysis were also responsible for the design, data collection and analysis of the original qualitative study; no additional researchers or authors were involved. Consistent with methodological recommendations for conducting qualitative secondary analysis [18], this analysis was guided by a distinct purpose and research question that differed from the original study.

### Participant recruitment

Maximum variation sampling was used to select eligible participants and ensure participant perspectives varied by age, gender, location of residence, employment and rare cancer type. People eligible to participate were [1] adults (≥ 18 years) currently living with a rare cancer, who received their diagnosis during adulthood (≤ 5 years ago), and [2] able to communicate fluently in English. Participants were recruited through Rare Cancers Australia’s (RCA) internal database of rare cancer patients. Participants received a Participant Information and Consent Form (PICF) that outlined the study purpose and procedure, as well as an online link to a survey to collect demographic information. All invitations, responses and interviews were managed by one researcher (TF). Participant recruitment ceased when no new themes relevant to the original study were obtained from the interviews.

### Data collection

Semi-structured interviews were conducted via Zoom video conferencing by one researcher (TF). Participant consent was reconfirmed verbally at the commencement of each interview. On the completion of each interview, one member from RCA’s Patient Support Team contacted the participant to provide support and address any distress experienced because of participation.

An interview guide (Appendix) was co-developed with RCA’s Patient Advisory Board, a consumer-led group of people affected by rare cancers, to ensure interview questions were appropriate and meaningful to participants. Interview questions focused on participant information needs, satisfaction with information received and preferences for information delivery; no questions were specifically asked about participants’ experience with illness uncertainty. The original interviews contained extensive and rich accounts of illness uncertainty amongst rare cancer survivors, indicating that the dataset was sufficient to support a focused secondary analysis. Researchers agreed that conducting a secondary analysis would avoid additional burden that conducting a new interview study would place on this vulnerable population group. Consequently, the research team conducted a secondary qualitative analysis to explore survivors’ experiences with illness uncertainty, a theme that was present but not fully explored in the original analysis. For the current analysis, all survivor interviews (*n* = 20) from the original study were analysed; no new data were collected.

### Data analysis

Interviews were audio recorded and transcribed verbatim for analysis. Uncoded interview data were independently analysed by one researcher (TF) applying thematic analysis, using NVivo 15 software. The analysis followed a theoretically informed, largely deductive orientation, applying key elements from Mishel’s RUIT used as a framework for identifying and interpreting interview data related to illness uncertainty. Specifically, survivor quotes were analysed and coded in respect to the form of uncertainty, stimuli frame, structure providers, and coping and adaptive strategies implemented by survivors.

## Results

### Participant characteristics

Participant characteristics are detailed previously [17]. In brief, 20 survivor interviews were conducted between November 2023 and February 2024, lasting between 23 min and 1 h and 31 min (mean 51 min). A total of six males (aged between 26 and 71 years) and 14 females (aged between 32 and 72 years) participated, with most residing in metropolitan areas (*n* = 17). One participant identified as Aboriginal and/or Torres Strait Islander. A total of eight rare cancer tumour types were identified; sarcoma was the most frequently reported diagnosis (*n* = 5), followed by thymic gland cancer (*n* = 4). Further information about participant characteristics can be found in Table 1.

**Table 1:** Participant characteristics (*n* = 19)

Characteristic	*n*	%
**Age**		
25–44 years 45–64 years ≥ 65 years	6 9 4	31.6 47.4 21.0
**Gender**		
Female Male	13 6	68.4 31.6
**Indigenous status**		
Do not identify Identify as Aboriginal and/or Torres Strait Islander	18 1	94.7 5.3
**Place of residence**		
Metropolitan Regional	17 2	89.5 10.5
**Level of education**		
Secondary school Apprenticeship/TAFE course University	4 4 11	21.0 21.0 57.9
**Cancer diagnosis**		
Adrenocortical carcinoma Blood cancer Gastrointestinal stromal tumour (GIST) NUT carcinoma Rare female genital cancer Sarcoma Thyroid cancer Thymic gland	1 2 2 1 2 5 2 4	5.3 10.5 10.5 5.3 10.5 25.3 10.5 21.1

### Key findings

#### Forms of uncertainty

The most prominent forms of uncertainty participants identified were ambiguity and receiving inconsistent or insufficient information (Table 2). Ambiguity was particularly acute when participants were undergoing investigation tests to confirm a diagnosis, where inconclusive test results heightened uncertainty. Participants described receiving insufficient information about their cancer type, potential treatment options and stage of disease. The lack of clarity left many participants unsure about what to tell family members, how to manage their finances and the potential impact on their employment. Ultimately, limited information and support heightened the sense of unknowing and confusion, where participants reflected on the emotional impact of this.

*“So, there was a period of time of not really knowing what was going on, or what was involved. And a lot of waiting. When you go on you get referred to someone, you sit there and you know that there’s something wrong. But you don’t know what it is, and no one can answer your question.” (P20, female, aged 33 years)*

**Table 2:** Rare cancer survivor’s experience with illness uncertainty in line with elements of the reconceptualised uncertainty in illness theory

Category of the RUIT	Subcategory	Definition	Participant quote
**Forms of uncertainty** The type of illness uncertainty a person experiences	Ambiguity	Uncertainty caused by the state of disease	*“Is it cancer? Is it not cancer like, how has it spread? Has it not spread?” (P01, female, aged 45 years)*
	Complexity	Uncertainty arising from intricate or complicated treatment or care procedures	*“There were just different markers and stuff that were coming up that made it difficult for them to pin down pathologically.” (P07, male, aged 26 years)*
	Inconsistent and/or inadequate information	Uncertainty arising from a lack of available information about the diagnosis and seriousness of the illness	*“It’s tough when, you're trying to get information about it yourself but the people and the resources that you’re trying to get the information from they don’t know, they don’t have anything for you.” (P17, male, aged 35 years)*
	Unpredictability	Uncertainty arising from an unclear course of disease and prognosis	*“There are so many things that come with it like no normal is new normal, that every time you think you’ve got the answer to where you’re at it changes. It either goes backwards or it goes forward or it goes sideways, or it does all of them at the same time.” (P06, male, aged 53 years)*
**Stimuli frame** The antecedents of uncertainty, consisting of the illness-related cues that a person interprets	Event familiarity	The degree to which a situation is repetitive or includes recognisable cues that are familiar to the person. A lack of prior experience, or unfamiliarity, leaves a person unsure and heightens uncertainty	*“…because you don’t know what’s going on. And you’re scared. You’re like ooh something seriously wrong, but you don’t seem to know what we do about it.” (P14, female, aged 47 years)*
	Event congruence	The consistency between a person’s expectations and their actual experience. When experiences do not meet expectations, it is more difficult to determine next steps, heightening uncertainty	*“I was told that I’d be catching a ball at six weeks and that’s far from the reality… in terms of thinking that rehab would be smooth sailing, it’s not been like that at all.” (P11, female, aged 34 years)*
	Symptom pattern	The degree to which symptoms are consistent and predictable. A consistent symptom pattern allows a person to anticipate events and lowers uncertainty	*“I had no idea what was really going on other than I had a mass.” (P01, female, aged 45 years)*
**Structure providers** Resources that assist the person in interpreting elements of the stimuli frame. Structure providers either directly or indirectly influence the stimuli frame and thereby a person’s appraisal of their situation and illness uncertainty	Credible authority	The degree of trust and confidence a person has in their healthcare provider/s (i.e. nurses and physicians)	*“I feel if the specialists don’t know anything about what you’ve just been diagnosed with, they shouldn’t be talking to you about it. They should refer you to someone that may know about it. Because they just increase that fear into you.” (P18, female, age unknown)*
	Social support	Family, friends, peers and caregivers who offer emotional, informational or practical support and influence the person’s interpretation of their situation	*“it really makes a difference for people to feel, somebody else’s been through this or is going through this, or it’s just started and I can help them.” (P09, female, aged 72 years)*
	Education	A person’s knowledge that helps to provide meaning and context to a situation	*“… you can choose not to take on the information if you don’t want it, but you know it arms you and prepares you for what’s next. It helps you to keep going. Knowing what the steps are coming to you, rather than this fear of not knowing anything. What it’s gonna be. That’s scary.” (P16, female, aged 49 years)*
**Cognitive capacity**		A person’s ability to process information, where limited capacity reduces a person’s ability to understand elements of the stimuli frame (i.e. event familiarity, event congruence and symptom pattern) [1]	*“A lot of it became really like a lot of loud background noise for me in the situation, sleep deprived, mentally stressed, physically exhausted, unable to walk. Spinal surgery, multiple cancers. It was like I had to kind of choose the information that I’m gonna concentrate on.” (P06, male, aged 53 years)*
**Appraisal** How a person interprets the meaning of uncertainty	Danger	When uncertainty is viewed as a threat or harmful, which leads to elevated anxiety, depression. A danger appraisal leads to coping strategies that reduce the uncertainty, such as information seeking and managing the emotion response	
	Opportunistic	When uncertainty is viewed as an opportunity, a positive outcome can be achieved and hope is magnified. An opportunistic appraisal leads to coping strategies that maintain the uncertainty, such as ignoring new stimuli that may change the view that uncertainty is positive	
**Coping strategy** How a person manages their illness uncertainty	Self-seeking information	The extent to which a person obtains information about their disease, treatment options, etc. to better understand their situation	*“I’m used to researching and I did extensive research myself, long before they came out with the diagnosis.” (P11, female, aged 34 years)*
	Self-organisation	The process by which a person constructs new meaning and normalcy in their daily life to live with their illness uncertainty	*“… a diagnosis like this is life changing so it makes you really reassess what your priorities are.” (P15, male, aged 52 years)*
**Adaptation** How a person accepts illness uncertainty as continuous and an inherent part of living with chronic disease	Acceptance	Part of the adaptation process, acceptance refers to a person’s ability to acknowledge their illness uncertainty and accept it as part of their daily life	*“I made peace with it very early on in the first couple of weeks so and I think I think one of the reasons I’m still here now is because I did actually accept the whole situation. I’m not afraid of death. It comes to all of us eventually anyway.” (P06, male, aged 53 years)*
	Gratitude	Part of the adaptation process, gratitude refers to a person’s appreciation of their situation and positive outlook on life	*“I’m very thankful that I’m still here. I’m very thankful I’ve had the extra time with family.” (P06, male, aged 53 years)*
	Probabilistic thinking	The person views illness uncertainty as natural and an inherent part of reality. Structure providers are important influencers of probabilistic thinking; when they support probabilistic thinking a person can more easily adapt to their illness uncertainty	*“I’d always take the positive in life. So even when you get this diagnosis I mean, you can count your blessings as well… Take the positive out of it and realise you can do that, you can, you’ve got some directions you’ve got a timeframe you can reassess your life.” (P12, male, aged 49 years)*

For many participants, uncertainty also took the form of complexity, as treatment options were often scarce or specialists with experience in the primary treatment were difficult to find. Many participants worried that their current treatment could lose effectiveness or become unavailable, leaving them with few or no alternatives.

*“…how long am I going to be able to access my current treatment for? If there’s not enough people that are taking the treatment, there’s no reason for them to keep making it even if there is a few of us there. There is a high chance that they will just stop, stop manufacture.” (P05, female, aged 32 years)*

#### Stimuli frame (i.e. sources of uncertainty)

Event (un)familiarity emerged as the primary stimuli-related source that contributed to participant’s illness uncertainty. Participants described being in unfamiliar situations where they, and at times their healthcare provider, lacked the knowledge or experience to understand what was happening. When seeking a diagnosis, participants found themselves navigating lengthy, unfamiliar processes which left them lacking a clear sense of direction. While a diagnosis provided temporary relief for some participants, uncertainty re-emerged when participants were forced to navigate the hospital system and multiple specialists, reintroducing event (un)familiarity.

*“Once I was diagnosed there were just quite a lot of unknowns. I just had lots of questions… how long I might be in hospital for... what the post operation care would consist of….” (P03, male, aged 71 years)*

Event (in)congruence was also evident when participants’ treatment or recovery experiences did not align with their expectations. Some participants explained how they did not receive important information about the recovery process or possible side-effects, which hindered their recovery and amplified uncertainty.

*“I’m in this false sense of ‘Oh it’s gonna be fine. I just take a tablet every night, and I’ll be sweet as, and I’ll go to work and my life will be normal’ and then I take the tablet and I, fifteen minutes after taking [it] on the first day I was bedridden.” (P01, female, aged 45 years)*

#### Structure providers (i.e. influencers of uncertainty)

Credible authority, through communication with healthcare providers, was consistently the most influential structure provider shaping participant’s experience of illness uncertainty. It was crucial that healthcare providers had the experience and expertise to manage participant’s rare cancer. During the diagnostic process, participants’ limited expertise led to reliance on their healthcare provider, trusting they were undergoing the appropriate tests and procedures to reach a diagnosis. When providers were unable to offer clear answers, honesty and trust were key to managing participants’ expectations, which helped survivors manage their illness uncertainty.

*“Let’s be real like you can only trust your oncologists. Your oncologist is the person that you see, that’s the number one specialist that you go to before you start treatment anyway. You go to them to find out what the plan is, they’re the expert in all of this... But you’re trusting them in their decisions. And you’re just hoping for the best.” (P17, male, aged 35 years)*

Social support was an important structure provider that influenced how uncertainty was experienced, providing hope in a turbulent time. However, this was mainly evident once the participant had completed treatment. Many participants described difficulties talking to family and friends, often due to a lack of understanding about the cancer. In these situations, participants felt alone and found peer support important, emphasising the value of connecting with others diagnosed with a similar cancer and having a shared experience.

*“It’s that sense of hope that you get when you’re able to speak to somebody else that’s been living with this cancer. It allows you to think, ‘okay, this is possible’. And that’s what you want to be able to hear. I can live with this cancer.” (P10, female, aged 55 years)*

#### Coping strategies and adaptation

The most common strategy for coping with illness uncertainty involved the participant searching for information themselves. Participants turned to online informational sources such as scientific journal articles for reputable information or social media to connect with others diagnosed with the same cancer. Information was seen as a tool for providing answers, enhancing understanding about one’s disease, and enabling participants to engage collaboratively with their treating team and advocate for themselves. Ultimately, seeking information helped participants regain a sense of control, prepare for the next steps and cope with their new reality.

*“I think the fact that I had done extensive research allowed me to handle the four days between diagnosis and surgery, ‘cause I knew what the surgery was, because from what I’ve I from the research that I had done.” (P11, female, aged 34 years)*

Participants also demonstrated self-organisation and finding a sense of normalcy, which helped them move forward amid ambiguity. Many participants described reprioritising aspects of life that were important to them, such as spending more time with family and friends. Continuing to work, talking about topics other than cancer and enjoying hobbies were ways in which participants dealt with their new reality.

*“…when you’re in that situation and being told some pretty heavy stuff you sort of holding onto as much normal as you can... It’s really disconcerting.” (P13, female, aged 43 years)*

Finally, there was an overwhelming sense of acceptance, where participants demonstrated adaptation to their illness uncertainty. Expressions of gratitude emerged as a key component of this adaptation process. Overall, participants expressed positivity and a desire to support others in similar circumstances as significant aspects of their adaptation process, particularly amongst those who had completed treatment.

*“… you have to kind of experience the experience. If that makes sense. ‘Cause if you don’t experience and process it at the time it then stays with you for longer… I have to learn how to live with it. I have to learn how to engage with what this means as a person and not as a body.” (P20, female, aged 33 years)*

## Discussion

### Main findings

Our study aimed to explore how rare cancer survivors experience and manage uncertainty throughout their cancer experience, applying Mishel’s RUIT to interpret our findings. Overall, participants described a persistent sense of uncertainty that spanned their entire cancer experience. The most consistent forms of uncertainty experienced were ambiguity, followed by uncertainty related to inconsistent or insufficient information. The most common stimuli-related source of uncertainty discussed was event (un)familiarity, which evolved to event congruence when survivors were undergoing treatment. Credible authority, specifically, healthcare providers, emerged as the structure provider with the greatest influence on illness uncertainty. Social support was important following the completion of treatment. Strategies to cope with illness uncertainty mostly involved searching for information, shifting into acceptance and gratitude once the participant had completed treatment.

### Interpretation of findings

Ambiguity and inadequate information were identified as the most consistent forms of illness uncertainty across the cancer pathway, which was largely attributed to the rarity of the participant’s disease. Specifically, participants described inadequate information about their specific cancer type, stage of disease and treatment options. Previous research indicates that insufficient information heightens ambiguity and uncertainty [19–21]. As rare cancer patients report unmet information needs and a paucity of information support across the rare cancer pathway [8], survivors may need to rely on alternative ways of managing their ambiguity.

Event (un)familiarity was described as the most prominent stimulus-related source of illness uncertainty, with participants reporting challenges in dealing with new and unfamiliar situations. This is in line with previous research that has shown that event familiarity significantly reduces uncertainty amongst cancer patients [16]. Also, rare cancer patients are more likely to receive their diagnosis and treatment in different hospitals compared to common cancer patients [22], leading to significantly lower continuity of care [23] and reduced event familiarity.

Credible authority, specifically, trusted healthcare providers, was identified as the primary influence on participants’ sense of illness uncertainty. This is consistent with previous research reporting that empathetic and understandable communication from healthcare providers reduces patient uncertainty [3, 16, 25]. Our findings suggest that in ambiguous situations, having trust in the healthcare provider is critical. This was particularly evident when participants reflected on their diagnostic and treatment experiences, where reliance on clinical expertise alleviated ambiguity and substituted the paucity of information with trusted clinical guidance.

The complexity of receiving a rare cancer diagnosis, combined with a surplus of medical information and demand for rapid decision-making during these periods, could explain why survivors relied predominantly on their healthcare provider for support. However, difficulties in maintaining trust, arising from fragmented care and the absence of a consistent contact person [26], highlight the importance of continuity of care in managing illness uncertainty and for optimising clinical and psychosocial outcomes [24].

Participants also reflected on the importance of social support for reducing uncertainty once they had completed treatment. In line with the previous research of de Heus et al. [27], transitioning to posttreatment and survivorship is likely to involve a greater reliance on psychosocial and emotional support, where there is a lesser need for medical information and complex decision-making. However, this finding contrasts with studies that have examined cancer patient support more broadly, where social support was identified as critical during diagnosis and when making treatment decisions [28, 29]. Future research should clarify how these influencers operate in rare cancer.

Consistent with previous research, participants’ coping strategies shifted as they progressed through their cancer experience [4]. Information-seeking was the primary coping strategy when participants were newly diagnosed or commencing treatment, supporting evidence that information needs decrease over time [30]. Participants sought information from online sources, including the internet and social media platforms, a finding consistent with earlier studies [31–33]. Research in common cancers suggests that poor information provision is a significant predictor of illness uncertainty [30]. Our findings suggest that this relationship could be amplified in rare cancers, where limited information and expertise increases reliance on self-directed information-seeking. Finally, participants who had completed treatment reported adaptation to their uncertainty, expressing positivity, acceptance and gratitude for their experience. In line with prior research on the experience of advanced cancer patients, a positive attitude has been associated with lower psychological distress [4, 34].

### Strengths and limitations

To our knowledge, no research has explored the experience of uncertainty amongst rare cancer survivors, despite the challenging trajectory these patients face. This is the main strength of our study. Secondly, analysing the interview findings in line with an established theoretical framework strengthens the analysis by providing an interpretive lens through which to understand the data. There are several limitations that should be addressed.

Firstly, this is a secondary analysis of qualitative data that were not originally collected to examine illness uncertainty. Consequently, exact dimensions of uncertainty may not have been explored consistently across all participants, and the findings reflect participants’ spontaneous accounts of uncertainty rather than a comprehensive assessment of all elements of the RUIT or coping and adaptation strategies. Additionally, while the data aligned with key elements of Mishel’s RUIT, appraisal of uncertainty, including how it impacted participants, was not explicitly explored. Secondly, due the retrospective nature of the study, recall bias is likely. Thirdly, the interviews were conducted at a single timepoint likely underestimating fluctuations in uncertainty throughout the cancer path. Finally, due to the heterogeneity of rare cancers in combination with the small sample size, it was not feasible to analyse whether uncertainty varied across rare cancer types.

### Future research and clinical implications

The negative impact illness uncertainty can have on psychological outcomes, in an already vulnerable population group, emphasises the importance of future research exploring this issue further. Our findings highlight the dynamic nature of coping with illness uncertainty throughout a rare cancer diagnosis and suggest the need for phase-appropriate approaches to uncertainty management. Studies should measure rare cancer survivors’ levels of uncertainty at each stage of the cancer path, using validated measurements of uncertainty such as Mishel’s Uncertainty in Illness Scale for Adults (MUIS-A) [35], and compare these to the experiences of those diagnosed with a common cancer. This would highlight where supportive care services should be offered to bridge the gap between these two groups to improve equitable cancer care.

## Conclusion

Our study demonstrates that rare cancer survivors experience illness uncertainty throughout the cancer pathway. This uncertainty is shaped by ambiguity attached to diagnosis, inadequate information provision and unfamiliarity with both the cancer and related events. Healthcare providers play a central role in influencing illness uncertainty, particularly in the context of limited information. Healthcare providers can reduce uncertainty by prioritising continuity of care and establishing themselves as a trusted clinical contact for patients facing ambiguous situations. Our findings suggest that the stimuli-related sources of uncertainty, influencers on uncertainty and coping strategies evolve throughout the rare cancer experience. Future research should focus on phase-appropriate interventions measuring illness uncertainty across the rare cancer trajectory and compare to the experiences of common cancer patients to inform where greater supportive care is needed.

## Data Availability

Data sharing is not applicable to this article. Transcripts of survivor interviews contain personal data and are therefore not available.

## Appendix

Interview guide for rare cancer survivors

- Welcome and introductions
- Purpose of the interview and how data will be used
- Guidance for interviewee, including emphasising the voluntary nature of the interview
- Topics to discuss, including the following:
  o Changing needs throughout the cancer trajectory
  o Satisfaction with information provided throughout cancer diagnosis
  o Preferences for information delivery
  o Development of an intervention that improves unmet information needs
- Closing the interview
